# Properdistatin inhibits angiogenesis and improves vascular function in human melanoma xenografts with low thrombospondin-1 expression

**DOI:** 10.18632/oncotarget.12695

**Published:** 2016-10-15

**Authors:** Jon-Vidar Gaustad, Trude G. Simonsen, Lise Mari K. Andersen, Einar K. Rofstad

**Affiliations:** ^1^ Group of Radiation Biology and Tumor Physiology, Department of Radiation Biology, Institute for Cancer Research, Oslo University Hospital, Oslo, Norway

**Keywords:** malignant melanoma, thrombospondin-1, properdistatin, tumor vasculature, intravital microscopy

## Abstract

In this study, the effect of properdistatin, a novel peptide derived from the thrombospondin 1 (TSP-1) domain of properdin, was investigated in three melanoma xenograft models with different TSP-1 expression. The tumors were grown in dorsal window chambers and were treated with 80 mg/kg/day properdistatin or vehicle. Morphological parameters of the tumor vasculature were assessed from high resolution transillumination images. Blood supply time (i.e., the time required for arterial blood to flow from a supplying artery to downstream microvessels) and plasma velocities were assessed from first-pass imaging movies recorded after a bolus of fluorescence-labeled dextran had been administered intravenously. Gene and protein expression of TSP-1 were assessed with quantitative PCR and immunohistochemistry, respectively. Properdistatin treatment inhibited angiogenesis in low TSP-1 expressing tumors but did not alter the vasculature in high TSP-1 expressing tumors. In low TSP-1 expressing tumors, properdistatin selectively removed small-diameter capillaries, but did not change the morphology of tumor arterioles or tumor venules. Properdistatin also reduced blood supply times and increased plasma velocities, implying that the treatment reduced the geometric resistance to blood flow and improved vascular function.

## INTRODUCTION

There is substantial evidence that melanoma progression requires angiogenesis. The transition from the horizontal to the vertical growth phase, which represents a significant worsening of prognosis, has been shown to depend on neovascularization [1], and high microvascular density in the primary tumor has been shown to increase the probability of metastasis in preclinical melanoma models [2]. Most tumor cells produce and secrete several proteins that may stimulate or inhibit angiogenesis, and the rate of angiogenesis is regulated by a balance between angiogenic stimulators and inhibitors [3]. Several strategies have been developed to inhibit angiogenesis, including targeting of angiogenic stimulators or their receptors with monoclonal antibodies or tyrosine kinase inhibitors [4–6], and treatments with endogeneous angiogenic inhibitors or small peptides that mimic these inhibitors [7]. Some of these strategies have been evaluated in clinical trials, but none of the antiangiogenic treatments have been shown to improve survival for patients with malignant melanoma when used as monotherapy [8]. Currently the effect of antiangiogenic treatment in combination with conventional chemotherapy or immunotherapy is evaluated for patients with malignant melanoma [8].

The effect of conventional chemotherapy and immunotherapy may be significantly affected by the tumor microenvironment. Tumors with extensive hypoxia are more resistant to immunotherapy and some forms of chemotherapy, and poor blood supply may reduce the uptake of therapeutic drugs [9, 10]. The microenvironmental effects of antiangiogenic agents have been investigated in several preclinical models including models of ovarian carcinoma, breast carcinoma, prostate carcinoma, glioblastoma, neuroblastoma, and melanoma [4, 5, 11–13]. The antiangiogenic treatments have improved vascular function and oxygenation in some of these preclinical studies, and impaired blood supply and induced hypoxia in others. The reasons for these different effects are not well understood but may have significant impact on combination therapies where antiangiogenic agents are applied before or concurrent to conventional therapy [14].

In most of the clinical trials evaluating the effect of antiangiogenic treatment in patients with malignant melanoma, agents targeting the vascular endothelial growth factor A (VEGF-A) pathway have been used. It is possible that agents targeting other angiogenic pathways may be more effective for this patient group. Thrombospondin-1 (TSP-1) is a potent angiogenic inhibitor that is produced and secreted by many tumor types [7]. This endogeneous inhibitor induces apoptosis in endothelial cells by binding to the transmembrane receptor CD36, and this mechanism prevents endothelial cells from responding to a wide variety of angiogenic stimulators [15, 16]. Treatment with exogeneous TSP-1 has been shown to inhibit angiogenesis in preclinical studies, but is considered inappropriate for clinical therapy due to the large size and the diverse biological effects of full-size TSP-1 [17–19]. The antiangiogenic domain of TSP-1 is mainly localized to three TSP-1 type 1 repeats, and small peptides that mimic these repeats have been developed [20]. One such peptide, ABT-510, has been evaluated in clinical trials of renal cell carcinoma and soft tissue sarcoma [21, 22]. ABT-510 was well tolerated but did not induce significant effects in these cancers. Small peptides derived from the TSP-1 type 1 repeats of other protein families have also been presented [23]. These include wispostatin-1 derived from WISP-1 and properdistatin derived from the plasma protein properdin. Wispostatin-1 has been shown to inhibit ocular neovascularization whereas properdistatin has been shown to bind to the CD36 receptor and to inhibit angiogenesis and tumor growth in breast carcinoma xenografts [24, 25].

In the current study, we evaluated the effect of properdistatin treatment in three human melanoma xenograft models growing in dorsal window chambers. The melanoma models differed in TSP-1 expression, and we report that properdistatin treatment inhibited angiogenesis in tumors with low TSP-1 expression. In these tumors, the treatment selectively removed small-diameter vessels and improved vascular function. Properdistatin treatment did not alter the vasculature in tumors with high TSP-1 expression.

## RESULTS

**Figure 1:**
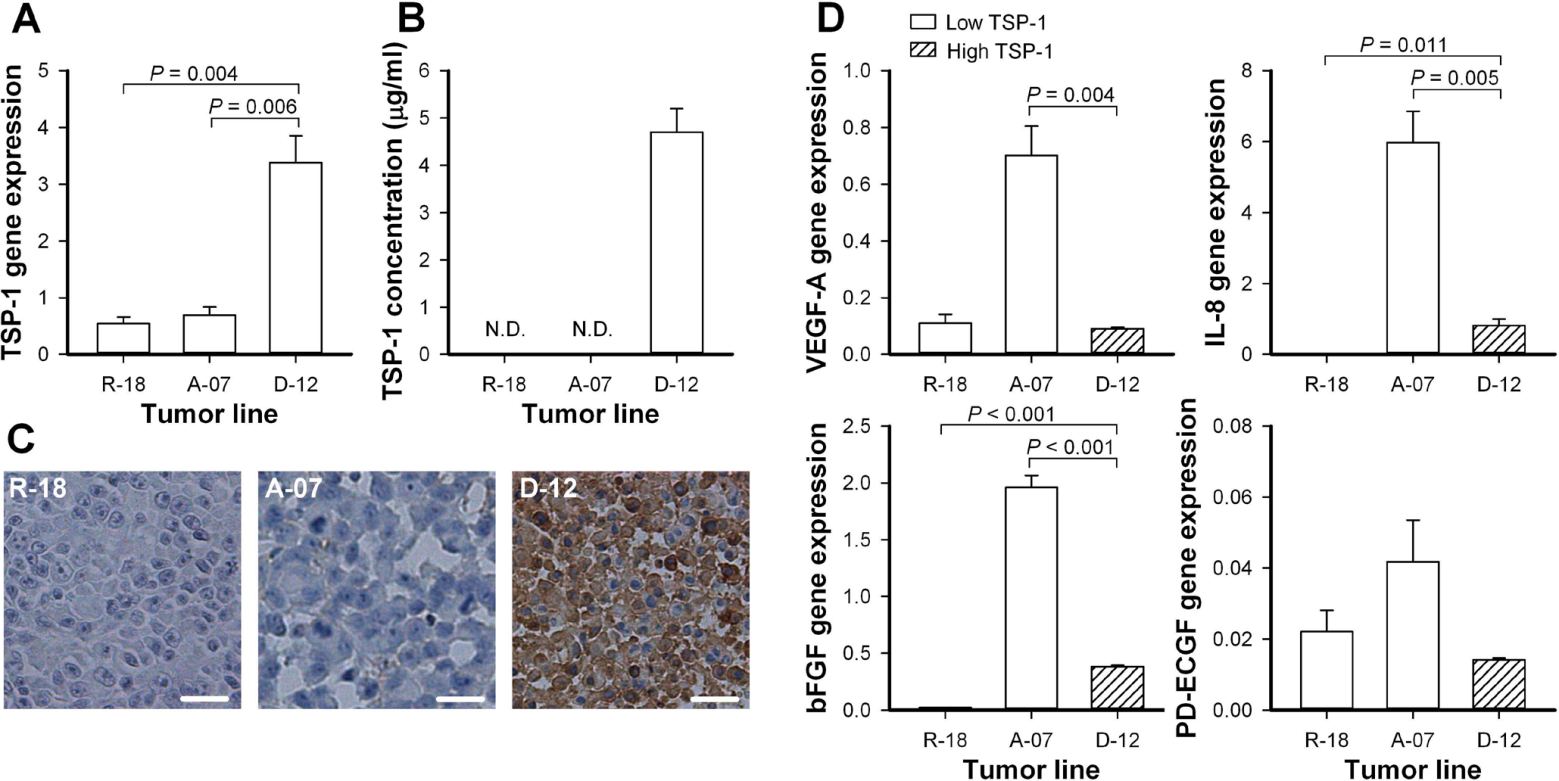
Expression of thrombospondin-1 and angiogenic stimulators (**A**) Normalized gene expression of thrombospondin-1 (TSP-1) in the R-18, A-07, and D-12 melanoma model. Columns, mean of three independent experiments; bars, SEM. (**B**) TSP-1 concentration in the blood plasma of untreated mice bearing R-18, A-07, or D-12 tumors. Columns, mean of 6 mice; bars, SEM; N.D., not detectable. (**C**) Immunohistochemical preparations of untreated R-18, A-07, and D-12 tumors stained with anti-TSP-1 antibody. Scale bars, 25 μm. (**D**) Normalized gene expression of vascular endothelial growth factor A (VEGF-A), interleukin 8 (IL-8), basic fibroblast growth factor (bFGF), and platelet-derived endothelial cell growth factor (PD-ECGF) in the R-18, A-07, and D-12 melanoma model. Columns, mean of three independent experiments; bars, SEM. Gene expressions were measured with quantitative PCR, was normalized to the mean expression of two housekeeping genes (GAPDH and ACTB), and multiplied with 100 (A and D).

The expression of TSP-1, VEGF-A, interleukin-8 (IL-8), basic fibroblast growth factor (bFGF), and platelet-derived endothelial cell growth factor (PD-ECGF) was assessed by quantitative PCR (Figure 1). The D-12 model showed significantly higher expression of TSP-1 than the R-18 and A-07 models (Figure 1A; D-12 *versus* R-18: *P* = 0.004, D-12 *versus* A-07: *P* = 0.006). The staining for TSP-1 was intense in immunohistochemical preparations of D-12 tumors, and substantially lower in R-18 and A-07 tumors (Figure 1C). TSP-1 was detected in the blood plasma of mice bearing D-12 tumors but was not detected in the blood plasma of mice bearing R-18 and A-07 tumors (Figure 1B). The TSP-1 expression was not correlated to the expression of the angiogenic stimulators VEGF-A, IL-8, bFGF, or PD-ECGF. Thus the expression of VEGF-A, IL-8, and bFGF was significantly higher in the A-07 model than in the D-12 model (Figure 1D, A-07 *versus* D-12, VEGF-A: *P* = 0.004, IL-8: *P* = 0.005, bFGF: *P* < 0.001), and similar or significantly lower in the R-18 model than in the D-12 model (Figure 1D, R-18 *versus* D-12, VEGF-A: *P* > 0.05, IL-8: *P* = 0.011, bFGF: *P* < 0.001). The A-07 and R-18 models did not differ from the D-12 model in the expression of PD-ECGF (Figure 1D, *P* > 0.05).

**Figure 2:**
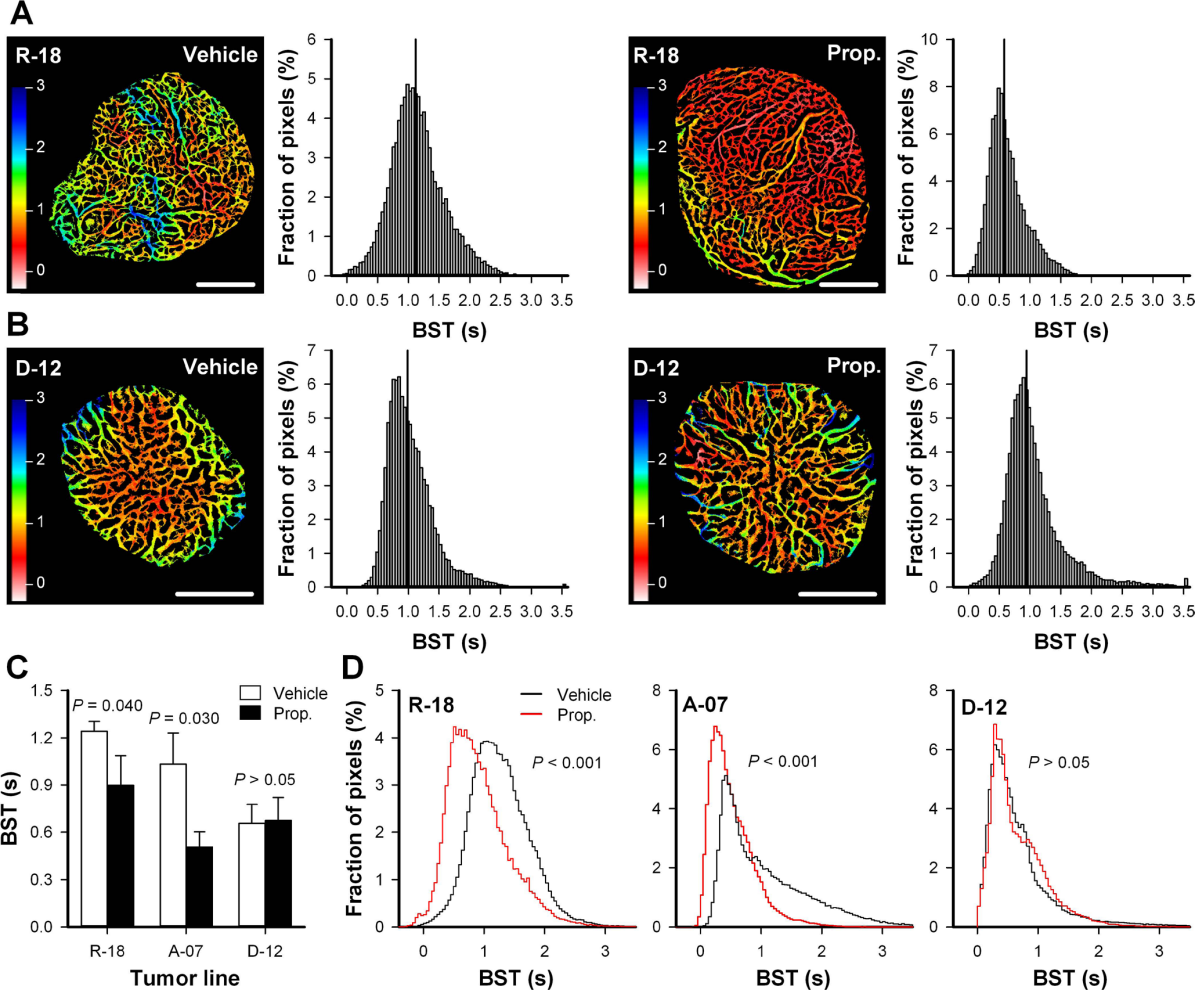
Vascular function (**A**–**B**) Blood supply time (BST) image and the corresponding BST frequency distribution of representative untreated and properdistatin-treated R-18 (A) and D-12 tumors (B). BST refers to the time required for arterial blood to flow from a supplying artery to downstream microvessels, and was calculated for every pixel within the tumor vasculature from first-pass imaging movies recorded after a bolus of 155 kD tetramethylrhodamine isothiocyanate-labeled dextran was injected intravenously. The BST images and the BST frequency distributions of the R-18 tumors (A) were produced from the first-pass imaging movies shown in Supplementary Movie S1. Color bars, BST scale in seconds; scale bars, 1 mm; vertical line, median BST. (**C**) Median BST in untreated and properdistatin-treated R-18, A-07, and D-12 tumors. Columns, mean of 4–7 tumors, bars, SEM. The median BST values of untreated and properdistatin-treated A-07 tumors have been reported previously [39] and are included for comparison with untreated and properdistatin-treated R-18 and D-12 tumors. (**D**) Total BST frequency distribution for untreated and properdistatin-treated R-18, A-07, and D-12 tumors. The total BST frequency distributions include all pixel values for all tumors within each treatment group. Tumors were treated with 80 mg/kg/day properdistatin or vehicle for four days.

The morphology and function of tumor vasculature was assessed by using intravital microscopy techniques. These techniques included recording of first-pass imaging movies after a bolus of fluorescence-labeled dextran was injectected intravenously. First-pass imaging movies of representative untreated and properdistatin-treated R-18 tumors are presented in Supplementary Movie S1. These movies show how the fluorescent bolus passes through the vascular networks, and illustrate that the bolus moved faster from the arterial to the venous side of the vascular networks in properdistatin-treated tumors than in untreated tumors. To quantify this qualitative observation, images and frequency distributions of the blood supply time (BST), i.e., the time required for arterial blood to flow from a supplying artery to downstream microvessels, were produced from first-pass imaging movies [26]. Figure 2A–2B shows BST images and the corresponding BST frequency distributions of representative untreated and properdistatin-treated R-18 and D-12 tumors. To compare untreated and properdistatin-treated tumors, median BST were calculated for individual tumors, and total BST frequency distributions were produced by including the pixel values for all tumors within treatment groups. Properdistatin-treated R-18 and A-07 tumors showed significantly lower median BST than untreated R-18 and A-07 tumors (Figure 2C; *P* = 0.040 for R-18, and *P* = 0.030 for A-07), and the BST frequency distributions of properdistatin-treated tumors differed significantly from those of untreated tumors for these tumor lines (Figure 2D; *P* < 0.001 for both R-18 and A-07). Untreated and properdistatin-treated D-12 tumors did not differ in median BST (Figure 2C; *P* > 0.05) or BST frequency distributions (Figure 2D; *P* > 0.05).

**Figure 3:**
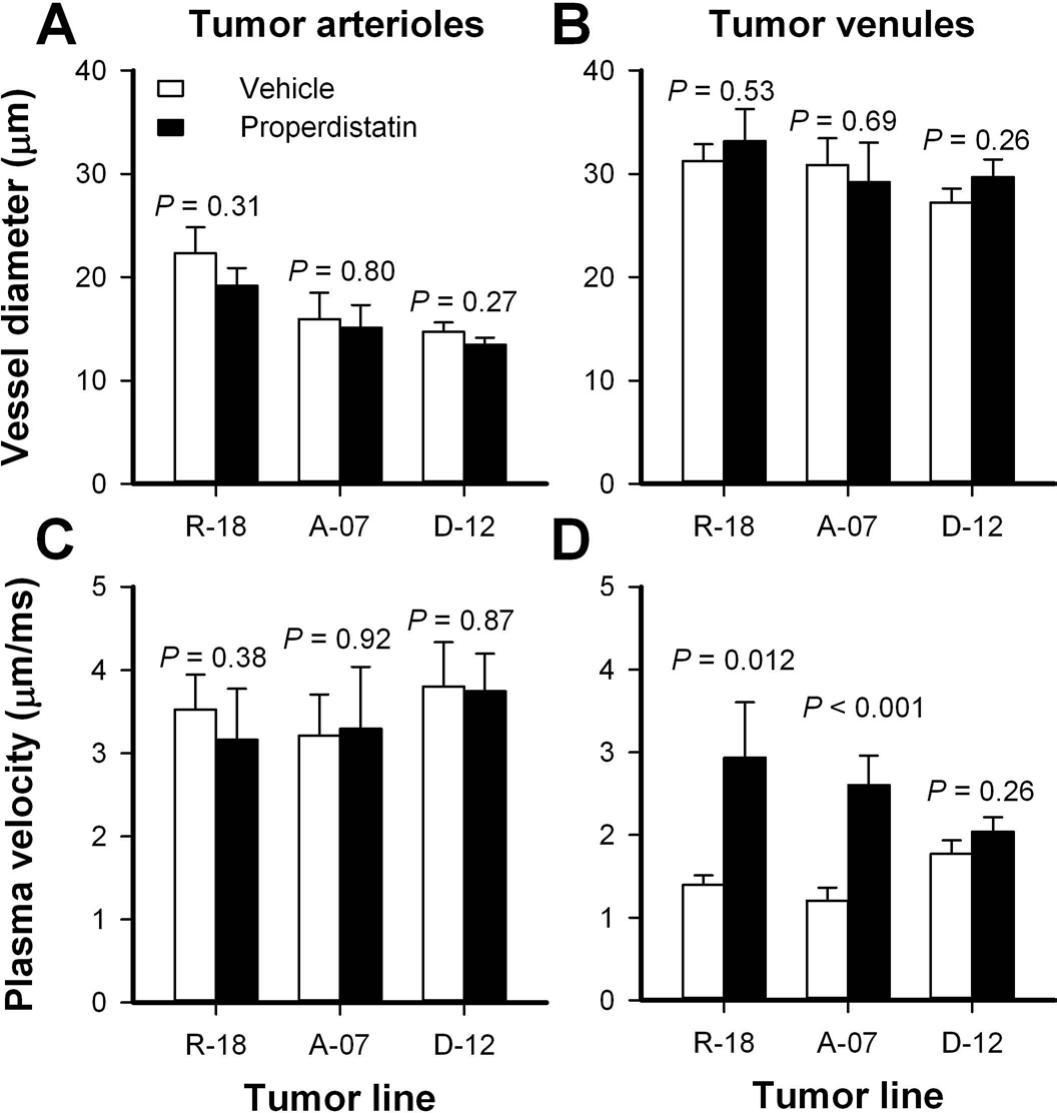
Tumor arterioles and venules Diameter and plasma velocity in tumor arterioles and tumor venules, in untreated and properdistatin-treated R-18, A-07, and D-12 tumors. Three tumor arterioles and 3 tumor venules were identified and measured in each individual tumor. Columns, mean of 4–7 tumors, bars, SEM. Tumors were treated with 80 mg/kg/day properdistatin or vehicle for four days. The diameters and plasma velocities of untreated and properdistatin-treated A-07 tumors have been reported previously [39] and are included for comparison with untreated and properdistatin-treated R-18 and D-12 tumors.

To investigate possible mechanisms for the properdistatin-induced reduction in BST, tumor arterioles (TAs) and tumor venules (TVs) were identified in the first-pass imaging movies and investigated in detail. The diameter of the TAs and the TVs did not differ between untreated and properdistatin-treated tumors in any of the tumor lines, suggesting that the treatment did not alter the morphology of TAs and TVs (Figure 3A–3B; *P* > 0.05). Untreated and properdistatin-treated tumors showed similar plasma velocities in TAs in all tumor lines (Figure 3C; *P* > 0.05). However, properdistatin-treated R-18 and A-07 tumors showed significantly increased plasma velocities in TVs compared to untreated R-18 and A-07 tumors (Figure 3D; *P* = 0.012 for R-18 tumors, and *P* < 0.001 for A-07 tumors). These observations suggest that properdistatin treatment induced morphological changes in the tumor capillary network (i.e. in tumor vessels that were supplied after the TAs and before the TVs) and that these changes resulted in both reduced BST and increased plasma velocities on the venous side of the tumor vasculature. Similar plasma velocities were found in TVs in both untreated and properdistatin-treated D-12 tumors (Figure 3D; *P* > 0.05) in accordance with the observation that properdistatin treatment did not affect BST in these tumors.

**Figure 4:**
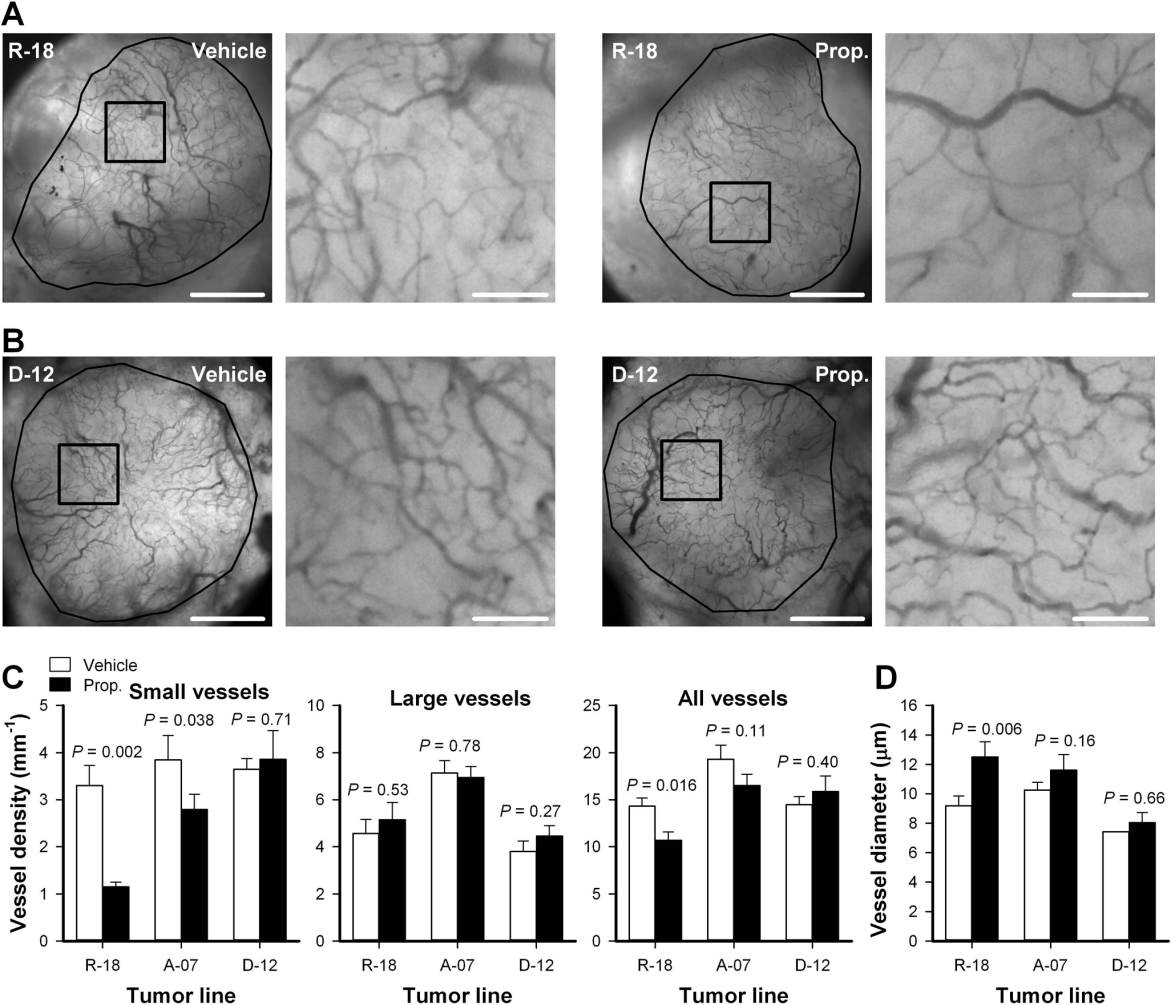
Vascular morphology (**A–B**) Intravital microscopy images of an untreated and a properdistatin-treated R-18 (A) and D-12 tumor (B). Tumor vessels were visualized by using transillumination and filters for green light. The images show the entire microvascular network of the tumors (left panels), and high-magnification image of the outlined region (right panels). Scale bars, 1 mm (left panels) and 100 μm (right panels). (**C–D**) Vessel density (C) and median vessel diameter (D) in untreated and properdistatin-treated R-18, A-07, and D-12 tumors. The density of small-diameter vessels (< 5 μm), the density of large-diameter vessels (> 15 μm), and the density of all vessels were measured separately and are shown in separate panels (C). Columns, means of 7–9 tumors; bars, SEM. Tumors were treated with 80 mg/kg/day properdistatin or vehicle for four days. The vessel diameter and vessel density of untreated and properdistatin-treated A-07 tumors have been reported previously [39] and are included for comparison with untreated and properdistatin-treated R-18 and D-12 tumors.

High-resolution transillumination images of the tumor vasculature were recorded to investigate how properdistatin treatment affected the morphology of tumor capillaries. Figure 4A–4B shows images of the tumor vasculature in untreated and properdistatin-treated R-18 and D-12 tumors, and Figure 4C–4D show quantitative data on vessel densities and vessel diameter. The density of small-diameter vessels (< 5 mm) was significantly reduced by properdistatin treatment in R-18 and A-07 tumors (Figure 4C; *P* = 0.002 for R-18, and *P* = 0.038 for A-07), whereas the density of large-diameter vessels (> 15 mm) was not changed by the treatment (Figure 4C; *P* > 0.05 for both R-18 and A-07). The reduction of small-diameter vessels was sufficiently large to induce a significant decrease in the overall vessel density and a significant increase in median vessel diameter in R-18 tumors (Figure 4C–4D; *P* = 0.016 for overall vessel density, *P* = 0.006 for vessel diameter), but not in A-07 tumors (Figure 4C–4D; *P* = 0.11 for overall vessel density, *P* = 0.16 for vessel diameter). In D-12 tumors, properdistatin treatment did not change vessel diameter and vessel densities regardless of whether the density of small-diameter vessels, the density of large-diameter vessels, or the density of all vessels were considered (Figure 4C–4D; *P* > 0.05).

**Figure 5:**
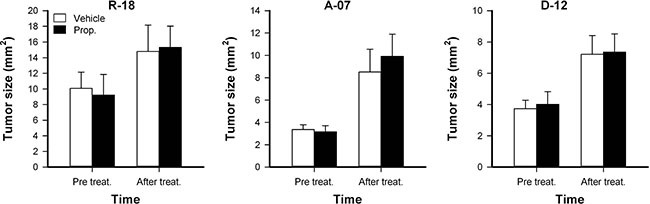
Tumor growth Tumor size in untreated and properdistatin-treated R-18, A-07, and D-12 tumors. Tumors were treated with 80 mg/kg/day properdistatin or vehicle for four days, and tumor size (i.e., tumor area) was calculated from the number of pixels showing GFP fluorescence before and after the treatment period. Columns, means of 7–9 tumors; bars, SEM.

Tumor size was measured before treatment and after the treatment was completed. Both untreated and properdistatin-treated tumors grew during the four-day treatment period, and properdistatin-treated tumors did not differ from untreated tumors in size at any time point (Figure 5; *P* > 0.05), implying that the short treatment did not affect tumor growth for any of the tumor lines.

## DISCUSSION

In most tumor types, TSP-1 expression is lost during malignant transformation [7]. Melanomas represent an exception from this rule because malignant melanoma cells show various expression of TSP-1 whereas melanocytes, their normal counterparts, do not show TSP-1 expression [27]. In the current study, properdistatin treatment inhibited angiogenesis in melanoma xenografts with low TSP-1 expression, and did not affect melanoma xenografts with high TSP-1 expression. In the R-18 and A-07 tumors, the properdistatin-induced increase in TSP-1 type 1 repeats was probably large compared to the amount of TSP-1 produced by the tumor cells, explaining why these tumors responded to properdistatin treatment. Properdistatin treatment probably resulted in a similar increase in TSP-1 type 1 repeats in D-12 tumors. However in the D-12 tumors, the increase was probably low compared to the amount of TSP-1 produced by the D-12 tumor cells. It is possible that properdistatin treatment may inhibit angiogenesis also in high TSP-1 expressing tumors if the properdistatin dose is increased. In accordance with this, exogeneous TSP-1 treatment has been shown to inhibit angiogenesis in D-12 tumors previously [28]. These observations imply that TSP-1-mimetic peptides may be developed to therapeutic drugs for patients with malignant melanoma, and that low TSP-1 expressing tumors are more likely to respond to such drugs than high TSP-1 expressing tumors.

The rate of angiogenesis is regulated by the ratio between angiogenic stimulators and inhibitors [3]. Tumors may be unresponsive to treatment with peptides that mimic endogenous angiogenic inhibitors if the tumors produce overwhelming amounts of angiogenic stimulators. We have previously shown that angiogenesis can be inhibited with antibodies against VEGF-A, IL-8, bFGF or PD-ECGF in the melanoma models included in the current study, and that the melanoma models differ in the secretion rate of these angiogenic stimulators [29]. However, the effect of properdistatin treatment was not correlated with the expression of the angiogenic stimulators because the responding A-07 model showed higher expression of the stimulators than the non-responding D-12 model, whereas the responding R-18 model showed similar or lower expression of the stimulators than the non-responding D-12 model. The A-07 model showed high expression of several angiogenic stimulators, but despite this, properdistatin treatment inhibited angiogenesis in A-07 tumors. This finding implies that TSP-1 type 1 repeats are highly potent angiogenic inhibitors.

Properdistatin treatment reduced BST and increased plasma velocities, implying that the treatment improved vascular function in melanoma xenografts with low TSP-1 expression. When laminar flow through a circular tube is assumed, the geometric resistance in a single vessel is inversely proportional to the vessel diameter in the fourth power [30]. Properdistatin treatment selectively removed small-diameter vessels which are expected to have a high geometric resistance to blood flow. The selective removal of small-diameter vessels probably reduced the geometric resistance in the vascular network and resulted in improved vascular function. This finding is consistent with a previous study from our laboratory which showed that treatment with exogenous TSP-1 reduces hypoxic fractions in melanoma xenografts [28].

Currently, clinical trials are evaluating whether antiangiogenic treatment in combination with immunotherapy or conventional chemotherapy can improve survival for patients with malignant melanoma [8]. The present study demonstrates that properdistatin treatment improves vascular function, suggesting that this treatment may increase the uptake of therapeutic drugs. Our study also demonstrates that properdistatin treatment does not affect all melanoma models, and thus careful monitoring of the tumor vasculature may be required if TSP-1 mimetic peptides are considered as neoadjuvant therapy to increase the uptake of immunotherapeutic or chemoteherapeutic drugs. We have previously demonstrated that dynamic contrast-enhanced and diffusion weighted magnetic resonance imaging is sensitive to treatment-induced changes in the tumor microenvironment induced by bevacizumab and sunitinib treatment in melanoma xenografts, suggesting that these non-invasive imaging techniques may be used to monitor the effect of TSP-1 mimetic peptides [31, 32].

The short treatment period (4 days) applied in the current study did not affect tumor size. This observation is consistent with our previous experience with exogenous TSP-1 treatment [28], and implies that short treatment periods with TSP-1 mimetic peptides are not likely to reduce tumor size in melanoma patients if the peptides are used as single treatment.

Some cancer patients show accelerated growth of pre-existing metastases after removal or curative treatment of the primary tumor. It has been hypothesized that primary tumors are capable of inhibiting neovascularization of metastases by secreting angiogenic inhibitors, and that removal of the primary tumor results in angiogenesis and tumor growth at metastatic sites [33]. One could thus speculate whether treatment with TSP-1 mimetic peptides may suppress metastases after high TSP-1 expressing primary tumors have been successfully cured. In accordance with this speculation we have demonstrated that treatment with exogeneous TSP-1 prevents accelerated metastasis after surgical removal or curative radiation treatment of high TSP-1 expressing melanoma xenografts [17].

In addition to properdistatin, several peptides have been derived from proteins containing TSP-1 type 1 repeats. These peptides include pentastatin-1 and chemokinstatin-1 which has been shown to inhibit angiogenesis and tumor growth in breast carcinoma xenografts, and wispostatin-1 which has been shown to inhibit ocular neovascularization [24, 25]. These peptides may also inhibit angiogenesis in melanoma xenografts and could possibly possess properties that make them more suitable for being developed to therapeutic drugs than properdistatin. Consequently, it is highly interesting to explore also these peptides in melanoma xenografts.

In summary, properdistatin treatment inhibited angiogenesis in human melanoma xenografts with low TSP-1 expression. The treatment selectively removed small-diameter vessels and improved vascular function. The current study suggests that TSP-1 mimetic peptides similar to properdistatin may be developed to therapeutic drugs for patients with malignant melanoma, and that such treatment may improve vascular function and increase the uptake of therapeutic drugs. Our study suggests that melanomas with low TSP-1 expression are more likely to be sensitive to TSP-1 mimetic peptides than melanomas with high TSP-1 expression.

## MATERIALS AND METHODS

### Tumor model

R-18, A-07, and D-12 human melanoma cells transfected with green fluorescence protein (GFP) obtained from our frozen stock were used in the present experiments [34]. Window chambers were surgically implanted in the dorsal skin fold of adult female BALB/c *nu/nu* mice, and tumors were initiated by implanting multicellular spheroids or tumor specimens with a diameter of 200 to 400 μm as reported earlier [35]. The animal experiments were approved by the Norwegian National Animal Research Authority and were done according to the Interdisciplinary Principles and Guidelines for the Use of Animals in Research, Marketing, and Education (New York Academy of Sciences, New York, NY).

### Anesthesia

Window chamber implantation and intravital microscopy examinations were carried out with anesthetized mice. Fentanyl citrate (Janssen Pharmaceutica, Beerse, Belgium), fluanisone (Janssen Pharmaceutica), and midazolam (Hoffmann-La Roche, Basel, Switzerland) were administered intraperitoneally (i.p.) in doses of 0.63 mg/kg, 20 mg/kg, and 10 mg/kg, respectively. After surgery, the mice were given buprenorphine (Temgesic; Schering-Plough, Brussels, Belgium) i.p. in a dose of 0.12 mg/kg to relieve pain.

### Properdistatin treatment

Properdistatin (Abgent, San Diego, CA) was dissolved in Hanks' balanced salt solution (HBSS). Mice were treated with 80 mg/kg/day properdistatin or vehicle (HBSS) by i.p. injection. The treatment started after the tumors were vascularized and lasted for 4 days. In a separate experiment, mice bearing A-07 tumors were treated with 20 mg/kg/day properdistatin. This low properdistatin dose has been shown to inhibit angiogenesis in breast cancer xenografts [25] but did not affect the tumor vasculature in A-07 tumors.

### Intravital microscopy

Intravital microscopy was performed as described in detail previously [35]. Briefly, mice with window chambers were fixed to the microscope stage during intravital microscopy, and the body core temperature was kept at 37 to 38°C by using a hot-air generator. Imaging was performed by using an inverted fluorescence microscope (IX-71; Olympus, Munich, Germany) and a black and white CCD camera (C9300–024; Hamamatsu Photonics, Hamamatsu, Japan). Tumor vasculature was visualized by using a ×4 objective lens, transillumination, and filters for green light, and images of the vasculature with a pixel size of 3.7 × 3.7 mm^2^ were recorded. To study the function of tumor vasculature, first-pass imaging movies were recorded after a 0.2 mL bolus of 50 mg/mL tetramethylrhodamine isothiocyanate-labeled dextran (TRITC; Sigma Aldrich, Schnelldorf, Germany) with a molecular weight of 155 kDa was injected into the lateral tail vein. First-pass imaging movies were recorded at a frame rate of 22.3 frames per second and a pixel size of 7.5 × 7.5 μm^2^ by using a ×2 objective lens and appropriate filters for TRITC.

### Analysis of vascular function and morphology

Two-dimensional projected vascular masks were produced from the movies recorded with the ×2 objective lens as described previously [35]. Blood supply time (BST) images were produced by assigning a BST value to each pixel of the vascular masks. The BST of a pixel was defined as the time difference between the frame showing maximum fluorescence intensity in the pixel and the frame showing maximum fluorescence intensity in the main tumor supplying artery, as described in detail previously [26]. Plasma velocities in tumor arterioles (TAs) and tumor venules (TVs) were calculated from the time lag in maximum fluorescence intensity along the vessel segments [36]. Mean TA plasma velocity, mean TV plasma velocity, mean TA diameter, and mean TV diameter were calculated from 3 TAs and 3 TVs in each tumor. Vessel density (i.e., total vessel length per mm^2^ tumor area) and vessel diameter were computed from manually produced vascular masks from transillumination images recorded with the ×4 objective lens as described previously [37].

### Quantitative PCR

RNA isolation, cDNA synthesis, and quantitative PCR were performed as described in detail previously [38]. Briefly, gene expression was assessed by using the RT^2^ Profiler PCR Array Human Angiogenesis (PAHS-024A) from SABiosciences (Frederick, MD). Real-time PCR was performed on an ABI 7900HT Fast Real-Time PCR instrument (Applied Biosystems, Carlsbad, CA). Each tumor line was run in three biological replicates. Glyceraldehyde-3-phosphate dehydrogenase (GAPDH) and β-actin (ACTB) were used as normalization genes because these housekeeping genes showed stable expression across the melanoma lines studied here. Thus, each replicate C_T_-value was normalized to the mean C_T_-value of GAPDH and ACTB (ΔC_T_ = C_T_^gene of interest^ – C_T_^mean of GADPH and ACTB^).

### ELISA

Blood plasma level of TSP-1 was measured by using commercial ELISA kits according to the instructions of the manufacturer (Chemicon International, Temecula, CA). Blood was collected from tumor-bearing mice by heart puncture. Plasma samples were prepared and analyzed promptly.

### Immunohistochemistry

The expression of TSP-1 in tumors was studied by immunohistochemistry, using an avidin–biotin peroxidase method. Tumors were fixed in phosphate-buffered 4% paraformaldehyde. Anti-human TSP-1 rabbit polyclonal antibody (TSP Ab-8; NeoMarkers, Fremont, CA) was used as the primary antibody, and hematoxylin was used for counterstaining.

### Statistical analysis

Statistical comparisons of data were carried out by the paired Student's *t* test when the data complied with the conditions of normality and equal variance. Under other conditions, comparisons were done by nonparametric analysis using the Mann-Whitney rank sum test. Probability values of *P* < 0.05 were considered significant. The statistical analysis was performed by using the SigmaStat statistical software (SPSS Science, Chicago, IL).

## SUPPLEMENTARY MATERIALS MOIVE




