# Supplementary material for: Properdistatin inhibits angiogenesis and improves vascular function in human melanoma xenografts with low thrombospondin-1 expression
Source: Oncotarget. 2016 Oct 15;7(47):76806–15. doi: 10.18632/oncotarget.12695 (PMC5363551; doi:10.18632/oncotarget.12695)
Supplement: Supplementary file 1 [file oncotarget-07-76806-s001.pdf]

Properdistatin inhibits angiogenesis and improves vascular function in human melanoma xenografts with low thrombospondin-1 expression

Supplementary Materials

Supplementary Movie S1: First pass imaging movies. First-pass imaging movies of a representative untreated R-18 tumor (left) and a representative properdistatin-treated R-18 tumor (right). The movies illustrate how a bolus of 155 kD tetramethylrhodamine isothiocyanate-labeled dextran (TRITC-dextran) moves through the vascular network of the tumors. Tumor arterioles are supplied at similar time points in both tumors. However the capillary network is supplied more efficient in the properdistaitn-treated tumor, and compared with the untreated tumor, the draining tumor venules are filled with TRITC-dextran earlier in the properdistaitn-treated tumor. The movies last for 5.8 s and were recorded with a temporal resolution of 44.8 ms and a pixel size of 7.5 × 7.5 μm^2. Tumor area is delineated by a solid black line. Scale bar, 1 mm. The BST image and the corresponding BST frequency distribution of these tumors are shown in Figure 2A. See Supplementary_Movie_S1
